# US Pediatric Emergency Department Visits for Mental Health Conditions During the COVID-19 Pandemic

**DOI:** 10.1001/jamanetworkopen.2021.8533

**Published:** 2021-04-30

**Authors:** Polina Krass, Evan Dalton, Stephanie K. Doupnik, Jeremy Esposito

**Affiliations:** 1 Division of Adolescent Medicine, Department of Pediatrics, Children’s Hospital of Philadelphia, Philadelphia, Pennsylvania; 2 National Clinician Scholars Program, University of Pennsylvania, Philadelphia; 3 Leonard Davis Institute of Health Economics, University of Pennsylvania, Philadelphia; 4 Division of General Pediatrics, University of Pennsylvania, Philadelphia; 5 PolicyLab, Center for Pediatric Clinical Effectiveness, Children’s Hospital of Philadelphia, Philadelphia, Pennsylvania; 6 Perelman School of Medicine, University of Pennsylvania, Philadelphia; 7 Division of Emergency Medicine, Children’s Hospital of Philadelphia, Philadelphia, Pennsylvania

## Abstract

This cross-sectional study describes the changes in the demographic characteristics and clinical outcomes of pediatric emergency department (ED) visits for mental health conditions during the COVID-19 pandemic.

## Introduction

The mental health (MH) of youth in the United States has been negatively impacted by the COVID-19 global pandemic.^1,2^ Containment measures, including restrictions and school closures, have been associated with the development and exacerbation of pediatric MH disorders.^3^ Pediatric emergency departments (EDs) have served an increasing role in assessing and triaging children with MH conditions over the past decade,^4^ and the COVID-19 pandemic has changed the system of pediatric MH care delivery.^5^ Therefore, we conducted this cross-sectional study to describe the changes in the demographic characteristics and clinical outcomes of pediatric ED visits for MH conditions during the COVID-19 pandemic.

## Methods

We collected data from the electronic medical record of 11 490 patients aged 5 to 24 years presenting to a tertiary children’s hospital ED between January 1, 2018, and January 1, 2021, with a MH diagnosis,^6^ MH chief concern, positive MH screening test result, MH evaluation, or requirement of physical restraint or safety observation. We compared the characteristics of ED MH visits before (from January 2018 to March 2020) and during (from April to December 2020) the COVID-19 pandemic. April 1, 2020, was used to separate these time periods because local and state restrictions were initiated during the final week of March 2020. Using multivariable logistic and linear regression, we evaluated for associations between presentation during the COVID-19 pandemic and patients’ odds of hospital admission and ED length of stay, respectively. Analyses were adjusted for race, ethnicity, sex, age, and insurance type. We evaluated ED length of stay separately for admitted and discharged patients. Analysis was performed using Stata, version 16 (StataCorp). We followed the Strengthening the Reporting of Observational Studies in Epidemiology (STROBE) reporting guideline for cross-sectional studies. The study was determined to be exempt from full review by the Children’s Hospital of Philadelphia institutional review board, and the requirement for written informed consent was waived given the use of deidentified data.

## Results

There were 11 490 ED visits for MH conditions and 272 358 total ED visits during the study period (Table). Although the mean number of monthly ED MH visits significantly decreased during the COVID-19 pandemic (from 338.6 to 260.8 visits per month), the proportion of ED visits for MH conditions significantly increased (from 4.0% [338.6 of 8559.9] to 5.7% [260.8 of 4582.3]), as shown in the Figure. Patients with ED MH visits during the COVID-19 pandemic were significantly more likely to be female (56.8% [5195 of 9143] to 62.6% [1469 of 2347]), White (34.8% [3176 of 9118] to 39.5% [924 of 2341]), and older than 12 years of age (70.7% [6465 of 9143] to 77.6% [1822 of 2347]) and were more likely to have commercial health insurance (40.3% [3686 of 9143] to 44.7% [1050 of 2347]). There was no significant difference in the proportion of patients identifying as Hispanic or Latinx (8.7% [795 of 9123] to 7.7% [181 of 2344]). Patients with MH conditions presenting during the COVID-19 pandemic more frequently required admission to the hospital (42.9% [3926 of 9143] to 52.7% [1236 of 2347]) and had higher adjusted odds of admission (adjusted odds ratio, 1.4; 95% CI, 1.28-1.54). The adjusted ED length of stay was shorter during the COVID-19 pandemic for both patients admitted for MH conditions (16.6 minutes shorter; 95% CI, 5.0-27.9 minutes shorter) and patients with MH conditions discharged from the ED (10.3 minutes shorter; 95% CI, 1.6-18.8 minutes shorter). The adjusted hospital length of stay for patients admitted for MH conditions was longer during the COVID-19 pandemic (3.4 days longer; 95% CI, 2.5-4.3 days longer).

**Table.** Characteristics of Pediatric Emergency Department Visits for Mental Health Conditions Before and During the COVID-19 Pandemic, 2018-2020

Characteristic	Patients, No. (%)	
	January 2018 to March 2020	April 2020 to December 2020
Visits for MH conditions, No.	9143	2347
Overall visits, No.	231 117	41 241
ED utilization (monthly)		
Visits for MH conditions, mean (SD), No.	338.6 (63.0)	260.8 (54.9)
Overall visits, mean (SD), No.	8559.9 (1176.2)	4582.3 (851.8)
Visits for MH conditions, mean (SD), %	4.0 (0.4)	5.7 (0.4)
ED length of stay, median (IQR), h		
Admitted patients with MH conditions	5.7 (4.4-7.6)	5.5 (4.2-7.3)
Discharged patients with MH conditions	3.9 (2.9-5.3)	3.9 (2.9-5.2)
Hospital length of stay of patients with MH conditions, median (IQR), d	2.7 (1.0-7.9)	4.9 (1.8-10.2)
Demographic characteristics		
Age, mean (SD), y	13.7 (3.6)	14.3 (3.4)
Adolescent patients^a^	6465 (70.7)	1822 (77.6)
Female patients	5195 (56.8)	1469 (62.6)
Male patients	3948 (43.2)	877 (37.4)
Hispanic or Latinx patients	795 (8.7)	181 (7.7)
Black patients	4631 (50.8)	1056 (45.1)
White patients	3176 (34.8)	924 (39.5)
Insurance type		
Commercial	3686 (40.3)	1050 (44.7)
Government	5310 (58.1)	1271 (54.2)

Abbreviations: ED, emergency department; IQR, interquartile range; MH, mental health.

^a^ Aged 13 years or older.

**Figure.**
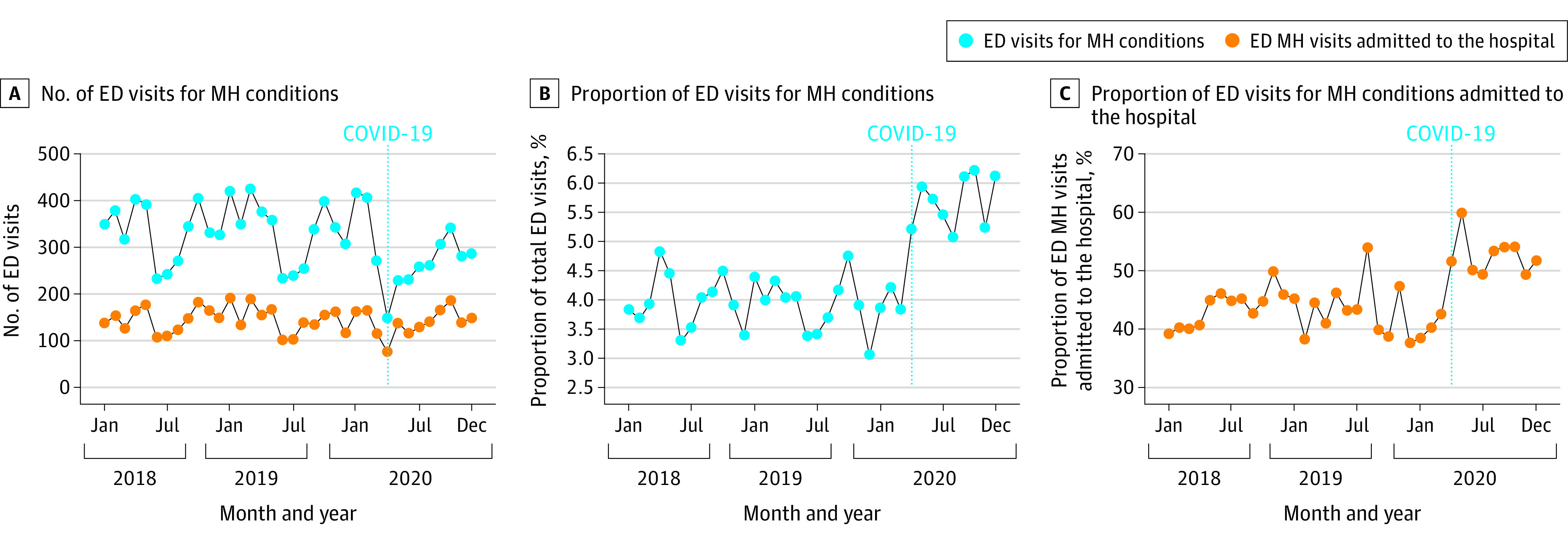
Monthly Pediatric Emergency Department (ED) Visits and Admissions for Mental Health (MH) Conditions, 2018-2020

## Discussion

The proportion of ED visits for MH conditions increased during the COVID-19 pandemic despite a decrease in the monthly mean number of ED visits for MH conditions. Patients with MH conditions presenting for ED visits since the onset of the pandemic have been more likely to require admission and have had longer admissions. The COVID-19 pandemic continues to place novel stressors on the provision of pediatric MH care. Our findings may reflect challenges in disposition to definitive MH care and may suggest a scarcity of MH treatment resources.

The limitations of this study include our inability to account for the complexity of presenting MH conditions and the limited generalizability to non-children’s hospitals. The findings of this cross-sectional study nevertheless underscore the need for increased pediatric MH services.
